# The effect of phytoestrogens (*Cimicifuga racemosa*) in combination with clomiphene in ovulation induction in women with polycystic ovarian syndrome: A clinical trial study

**DOI:** 10.22038/AJP.2021.47998.2595

**Published:** 2022

**Authors:** Seyedeh Azam Pourhoseini, Maliheh Mahmoudinia, Mona Najaf Najafi, Fouad Kamyabi

**Affiliations:** 1 *Department of Obstetrics, Faculty of Medicines, Mashhad University of Medical Sciences, Mashhad, Iran*; 2 *Department of Obstetrics, Faculty of Medicines, Mashhad University of Medical Sciences, Mashhad, Iran*; 3 *Clinical Research Development Unit, Imam Reza Hospital, Mashhad University of Medical Sciences, Mashhad, Iran*; 4 *School of Medicine, Mashhad University of Medical Sciences, Mashhad, Iran*

**Keywords:** Polycystic ovarian, Cimicifuga racemose, Phytoestrogen, Ovulation induction, Clomiphene

## Abstract

**Objective:**

Phytoestrogens can be used as an alternative to clomiphene for ovulation induction in patients with polycystic ovarian (PCO). In this study, we evaluated the impact of phytoestrogens (*Cimicifuga racimosa*) in combination with clomiphene on the endometrium thickness and follicle number in women with PCO.

**Materials and Methods:**

This study was a prospective clinical trial conducted in the infertility research center of Milad Hospital in Mashhad, Iran, during 2016 and 2017 on 100 women with PCO syndrome. The patients were randomly divided into two equal groups. The patients in both groups were treated by clomiphene citrate 50 mg, twice a day for 5 days, from the second day of the menstrual cycle and for three consecutive cycle periods. The intervention group received *Cimicifuga racemosa *tablets 10 mg twice a day for 10 days from the second day of the menstrual cycle, in addition to the mentioned standard treatment. The two groups were compared in terms of the number and size of follicles and endometrial thickness on the ultrasound.

**Results:**

There was no significant differences between the number of medium (p=0.288), large (p=0.086), and total (p=0.288) follicles between the two groups. Also, no significant difference was observed in endometrial thickness between the two groups (p=0.227).

**Conclusion:**

As a result, adding *Cimicifuga racemosa* to clomiphene could not increase the endometrial thickness and the number of follicles in PCO patients.

## Introduction

Polycystic ovary (PCO) was first diagnosed in 1935 by Stein and Leventhal, and is the most common endocrinopathy in women of reproductive ages with about 6.5% prevalence worldwide (Kamel, 2013 ▶) PCO is currently accepted as a syndrome with a set of clinical signs and symptoms. However, there is still no comprehensive agreement on the diagnosis and definition of this syndrome (Saha et al., 2013 ▶). 

Women with PCO are at higher risk for cardiovascular diseases, cerebrovascular diseases, hypertension, dyslipidemia, myocardial infarction, glucose tolerance disorder, metabolic syndrome, and central obesity, compared to healthy women (de Medeiros et al., 2013 ▶). Anovulation is a key feature of the disease which occurs in 20-50% of cases with amenorrhea and 30% of cases with severe and irregular bleeding (Berek and Novak, 2012 ▶).

One of the methods to stimulate ovulation in the PCO is the use of clomiphene citrate, a weak synthetic estrogen that mimics estrogen-antagonistic activity when used at the usual pharmacological doses (Fritz and Speroff, 2011 ▶). 

Due to its structural similarity, clomiphene competes with endogenous estrogen to bind with estrogen nuclear receptors throughout the reproductive system. However, unlike estrogen, clomiphene binds with these receptors for a long time and thus interferes with receptor recovery, lowering the receptor concentration. The reduction of estrogen-negative feedback initiates natural compensatory mechanisms that alter the pattern of gonadotropin-releasing hormone secretion and increase the secretion of pituitary gonadotropins which in turn stimulate the ovarian follicular development (Berek and Novak, 2012 ▶).

The adverse effects of clomiphene on endocervix, endometrium, ovary, etc. have been described. Nevertheless, there is no convincing evidence to suggest that these effects have significant clinical consequences in most women (Berek and Novak, 2012 ▶).

Phytoestrogens are herbal estrogens derived from a variety of herbal products. Phytoestrogens are said to have poor effects on the endocrine system compared with the steroidal estrogens, but they have the potential to have both physiological estrogenic and anti-estrogenic effects )Becker et al., 2005 ▶). Phytoestrogens can bind with the estrogen receptors (ER) with a greater affinity for binding to estrogen receptors β than estrogen receptors α (ERα). Nonetheless, phytoestrogens are generally less potent than endogenous estrogens such as 17-beta estradiol (Bu and Lephart, 2005 ▶). The use of phytoestrogens with clomiphene citrate to reverse the anti-estrogenic effects of clomiphene on the endometrium, increases endometrial thickness and reduces the risk of miscarriage and higher pregnancy rates (Unfer et al., 2014 ▶).

*Cimicifuga racemosa* is one of the phytoestrogens that have estrogen-like effects and is a competitive antagonist for the endogenous estrogen. It stimulates the central system to secrete gonadotropin and this feature is used to induce ovulation in women with PCO. (Kamel, 2013 ▶). Several studies have examined the effect of phytoestrogens in improving the harmful effects of clomiphene on induction and ovulation. (Maged and Deeb, 2015 ▶; Kamel, 2013 ▶).

Owing to the prevalence of PCO and its associated infertility, and given that this infertility can affect different aspects of an individual’s life such as mental, psychological, and social aspects, we decided to develop a new, accessible, and appropriate method to stimulate ovulation in women so that by increasing the fertility rate, we can reduce the psychological burden of these patients to some degree. Therefore, this study aimed at examining the effect of phytoestrogens (*Cimicifuga racemosa*) in combination with clomiphene in ovulation induction in women with PCO.

## Materials and Methods

**Study design**

This study is a prospective clinical trial conducted in the infertility research center of Milad Hospital in Mashhad between 2016 and 2017 on the women with PCO. This study was approved by the ethics committee of the research deputy of Mashhad University of Medical Sciences (IR.MUMS.REC. 1396.8490), and it was registered in the clinical trial website of Health ministry of Iran (IRCT20141209020264N2).

**Participants**

**Figure 1:**
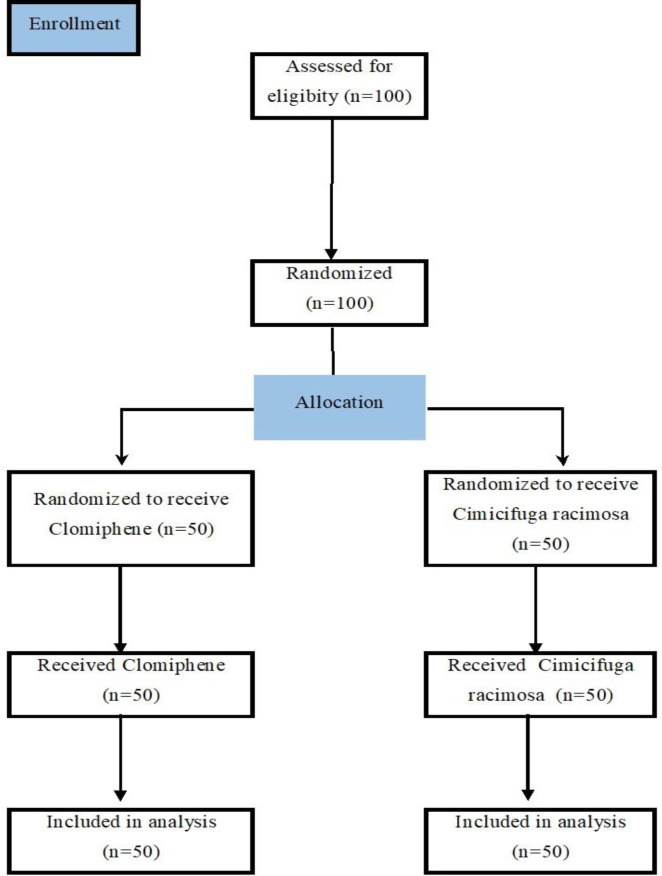
CONSORT flow diagram of the study

The participants of the study were 100 women with PCO whose age varied from 14 to 28 years old (Mean=25.09, SD=3.81). (Figure 1) Before entering the study, the participants were first provided with the necessary information about the research and their duties during the research process. Then, they were asked to complete the informed consent forms. The inclusion criteria were as follows: all women with PCO with a history of primary and secondary infertility who had normal hysterosalpingography and husband’s spermogram was normal, as well as being≤28 years old. The exclusion criteria included having cardiovascular disease, liver disease, and ovarian mass, as well as being>28 years old.

**Intervention**

At the beginning of the study, all participants were required to express their medical history. Moreover, they went through general examinations, pelvic examinations, laboratory examinations, and transvaginal ultrasound by a gynecologist to confirm the existence of the criteria for PCO. 

After the diagnosis of PCO and receiving the participants’ consent forms to take part in the study, each participant was given an envelope in which there was a green or a blue card. Through these cards, the subjects were randomly divided into two groups.  

The group who received the blue card was required to take clomiphene citrate 50 mg (produced by Iran Hormone Company) tablet twice a day for 5 days; they started using the drug from the second day of their menstrual cycle. The study was conducted on their three menstrual cycles. In the first cycle, they were asked to take the tablet. The second cycle was their rest period that is they did not take any drugs. In the third cycle, they did the same as the first cycle. The patients with the blue cards received no treatment other than standard treatment that was stated. However, the group with the green cards, in addition to the standard treatment that was mentioned for the first group, were required to take *Cimicifuga racemosa* 10 mg tablet (produced by Isfahan Goldaru Company) twice a day for 10 days. The same as the first group, they started taking the tablets from the second day of their menstrual cycle. 

The transvaginal ultrasound was performed on the 14th day of the cycles in which the patients took the drugs, to detect the number and size of mature follicles and to measure the endometrial thickness. When the size of one or two follicles reached 18 mm or more, Human chorionic gonadotropin (hCG) was prescribed. Then, they were recommended to have intercourse at the appointed time. 

The two groups were compared regarding the ultrasound characteristics (the number and size of the mature follicles and the endometrial thickness) before and after the treatment and chemical pregnancy (based on βHCG test). The study was a double-blind study; the patients did not know in which drug group they participated. The physician also did not know which patient belonged to which group. 

**Data analysis**

After data collection, the accuracy of the information was reviewed. Mean and standard deviation were used to describe the data, and if not normally distributed, the median and quadratic indices were used. The inferential statistical tests were also selected based on the normality conditions of the data. To examine the homogeneity of the two groups regarding their demographic information, Chi-Square tests were run. To compare the number of follicles between the two groups, Chi-Square test was used. To investigate the difference between the intervention and control groups concerning the endometrial thickness, an independent t-test was run. Data were analyzed using SPSS software version 16 and the significance level was set at 0.05. 

## Results

The demographic information of the two groups of the participants is displayed in Table 1. As the Table displays, there were no significant differences between the intervention and control groups regarding their jobs (χ2=0.102, p=0.749), the jobs of their spouses (χ2=1.333, p=0.248), their education (χ2=0.437, p=0.509), their previous abortion history (χ2=0.122, p=0.727), their previous stillbirth history (χ2=3.09, p=0.079), and their smoking status (χ2=0.102, p=0.749) (see Table 1). To compare the number of medium )10 mm to 15 mm (, large ) more

than 15 mm (and total follicles between the intervention and control groups, a Chi-Square test was conducted. As Table 2 demonstrates, there were no significant differences in the number of medium (χ^2^=1.131, p=0.288), large (χ^2^=2.941, p=0.086), and total (χ^2^=1.131, p=0.288) follicles between the two groups. To examine the difference between the two groups concerning the endometrial thickness, an independent t-test was run. The results revealed no statistically significant differences between the two groups of PCO women who received clomiphene alone or a combination of clomiphene and *Cimicifuga racimosa* (t=-1.085, p=0.281) (See Table 3).

**Table 1:** Comparison of the demographic information of the two groups

		Intervention	Control	Value .	
Participant job	Employed Housewife	5 45	6 44	0.102^a^	0.749^a^
The job status of the participant’s spouse	Freelance job Employee	35 15	40 10	1.333^a^	0.248^a^
Participants’ education level	Diploma and under diploma Above diploma	37 13	34 16	0.437^a^	0.509^a^
Previous abortion history	Yes No	5 45	4 46	0.122^a^	0.727^a^
Previous stillbirth history	Yes No	3 47	0 50	3.09	0.079^a^
Participant smoking status	Yes No	0 50	4 46		
Participant’s spouse smoking status	Yes No	8 42	11 39	0.585^a^	0.444^a^
Age (year)	Range Mean SD	14 24.740 3.691	18 25.440 3.944		

^a ^Pearson Chi-Square

**Table 2:** Comparison of the number of medium, large and total follicles between the intervention and control group

			Medium )10 to 15 mm (		Large< 15 mm		Total	
			No	Yes	No	Yes	No	Yes
Group	Intervention (Clomiphene and Cemifogul)	Number % Within- group	14 28.0%	36 72.0%	20 40.0%	30 60.0%	7 14.0%	43 86.0%
	Control (Clomiphene alone)	Number % Within- group	19 38.0%	31 62.0%	12 24.0%	38 76.0%	2 4.0%	48 96.0%
	χ^2^		1.131^a^		2.941^a^			
	p		0.288^a^		0.086^a^			

^a^ Pearson Chi-Square

**Table 3:** Comparison of the endometrial thickness between the intervention and control groups

	Group	N	Mean	SD	equality of means		
*Endometrial Thickness* mm	Clomiphene and Cemifogul	49	6.6653	2.21807	t	df	Sig. (2-tailed)
	Clomiphene alone	46	7.1087	1.71345	-1.085^a^	93^a^	.281^a^

^a ^t-test

## Discussion

In this double-blind clinical trial study conducted at Mashhad Infertility Center, we attempted to compare the endometrial thickness and the number of follicles in the women with PCO treated with either clomiphene alone or a combination of clomiphene and *Cimicifuga racemosa* . The findings of the research revealed no statistically significant differences between the two groups regarding their endometrial thickness or the number of their follicles. 

As Wuttke et al explained* Cimicifuga racemosa* performs its function through selective estrogen receptor modulator (SERM) mechanism (Wuttke et al., 2008 ▶). Phytoestrogens, either through activation of the ER with less antiestrogenic activity or through the mechanism of SERM, increase Follicle Stimulating Hormone (FSH) secretion or ameliorate the detrimental effects of clomiphene on granulosa cells, oocytes, endometrium and possibly cervical mucosa. These processes lead to maturation of follicles, thicker endometrium in a shorter time, and improved fertility rates (Wuttke et al., 2008 ▶).

The positive results of *Cimicifuga racemosa* in patients with PCO may be related to intra-ovarian androgen accumulation. The possible inhibition of aromatase expression by this herbal drug is one of the probable causes of the decrease in estrone (E1) and estradiol (E2) levels in the pre- and post-menopausal mammary tissue (Beck et al., 2003 ▶).

Although phytoestrogens have estrogen-like effects in *in vitro* and *in vivo* studies as well as on the endometrium of postmenopausal women (at a dose of 1500 mg/day), they act as a weak anti-estrogen substance in estrogen-rich environments such as PCO patients (Shahin and Mohammed, 2014 ▶). This anti-estrogenic effect at the clinical level has an antagonistic impact on the effects of high doses of estradiol on the uterine mucosa. As a result, it reduces the level of estradiol activity and improves endometrial receptivity. These effects can be seen in studies that added *Cimicifuga racemosa* to natural cycles or high estrogen levels such as intrauterine insemination (IUI) and In vitro fertilization (IVF) (Stute et al., 2007 ▶). In another study, it was found that high doses of phytoestrogens combined with clomiphene citrate eliminated the deleterious effects of clomiphene on endometrial thickness in women candidates for IUI, causing a thicker endometrium, reducing abortion rates, and increasing ongoing pregnancy in these patients (Unfer et al., 2014 ▶).

In the present study, there was no significant difference between the intervention and control groups in the number of large follicles or the total number of medium and large follicles. Moreover, there was no significant difference in the endometrial thickness between the two groups. There have not been many studies on the effect of phytoestrogens on PCO patients and therefore the results of this study cannot be compared and critically evaluated. However, in a study carried out by Maged et al. it was revealed that patients receiving phytoestrogen adding to clomiphene, had more the number of dominant follicles and an improvement in pregnancy rates. (Maged and Deeb, 2015 ▶). The difference between the results of this study and ours may probably be due to the different definitions of small, medium, and large follicles. In our study, the follicles with a diameter less than 10 mm were considered small, those possessing a diameter between 10 and 15 mm were medium, and those with a diameter of more than 15 mm were large. But, in Maged et al. study, the size of the follicles was less than 14 mm, 14-18 mm and greater than 18 mm.

However, in their study, the endometrial thickness was significantly higher in the group treated with clomiphene and phytoestrogens compared to the group treated by clomiphene alone. In contrast, in our study, there was no statistically significant difference between the intervention and control groups regarding the participants’ endometrial thickness.  Similar to the findings of Maged et al. the results obtained by Shahin et al. demonstrated that phytoestrogens in combination with clomiphene produced a thicker endometrium in PCO patients compared with those treated with clomiphene alone (Shahin and Mohammed, 2014 ▶). In a similar vein, Kamel, acquired the same results (Kamel, 2013 ▶). However, our findings are in contrast with those of the previous studies. Contemplating the possible reasons for such as a contrast, the researchers of the current study came across to the following reason: The difference might be plausibly justified by the fact that we used the Iranian sample of *Cimicifuga racemosa* with the Cimifogel brand, whereas in other studies mainly in Egypt, the German tablet with the brand name Klimadynon was used. It seems that further clinical trials can reveal the difference between the two types of drugs affecting PCO patients.

Yet, our research suffers from some limitations that should be addressed in future studies. One of the limitations of this study is the small sample size which was due to the feasibility issue. It is recommended to repeat the study with a larger sample size to see whether similar results would be obtained. Also, as the mechanisms underlying how phytoestrogens affect the PCO process are not yet known, it is recommended to evaluate and compare the serum levels of progesterone,  luteinizing hormone (LH), FSH, insulin, testosterone, estradiol, and estrone, concurrently. On the other hand, considering the discrepancy in the results of the present study with those of the previous ones, it is recommended to repeat the study in Iran , using the German (Klimadynon) of the tablet and compare its impact with the Iranian tablet (Cimifogel) to see whether the same results would be obtained. Moreover, this comparison might reveal the reasons why the findings of the current study differ from those of the previous ones. 

 In sum, based on the findings of this study, we can conclude that adding Cimifogel to clomiphene could not increase the endometrial thickness or the number of follicles in PCO patients.

## Conflicts of interest

The authors have declared that there is no conflict of interest.
